# Supplementary figures and images for: Hatching-Box: Automated in situ monitoring of Drosophila melanogaster development in standard rearing vials
Source: PLoS One. 2025 Sep 29;20(9):e0331556. doi: 10.1371/journal.pone.0331556 (PMC12478940; doi:10.1371/journal.pone.0331556)

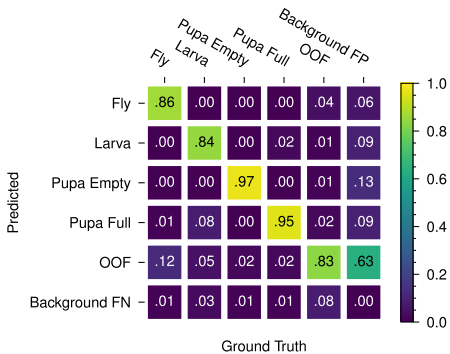

(a) YOLOv7-tiny

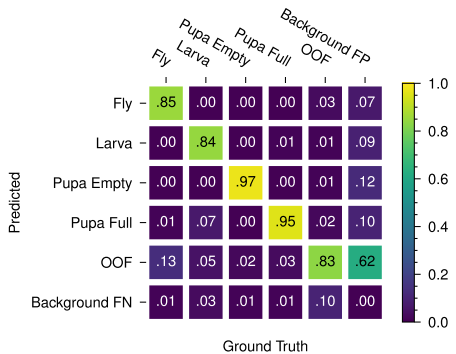

(b) YOLOv7

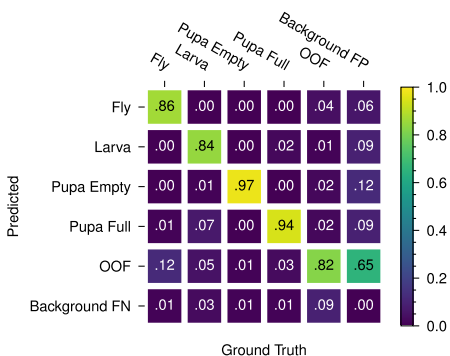

(c) YOLOv7-X

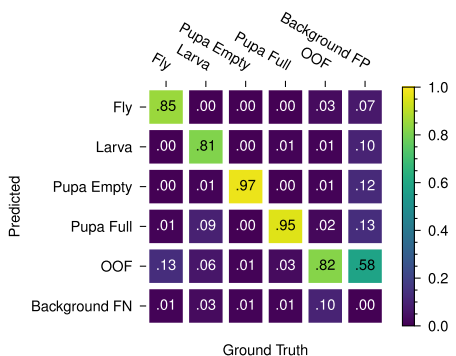

(d) YOLOv7-E6

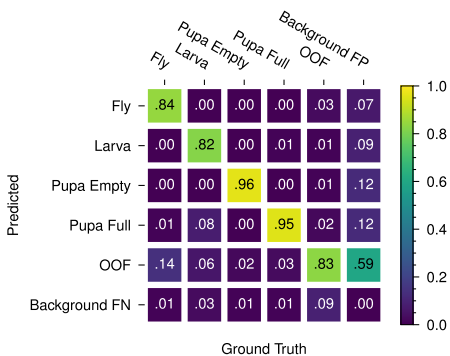

(e) YOLOv7-W6

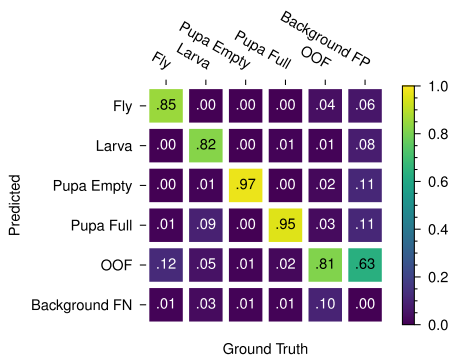

(f) YOLOv7-E6E

Supplement: S1 Fig — (PDF) [file pone.0331556.s001.pdf]

period

0  
1  
2  
3  
4  
5  
6  
7  
8  
9  
10  
11  
12

iso31

per\_0

per\_l

per\_s

0 12 24 36 48

0 12 24 36 48

0 12 24 36 48

0 12 24 36 48

Time (h)

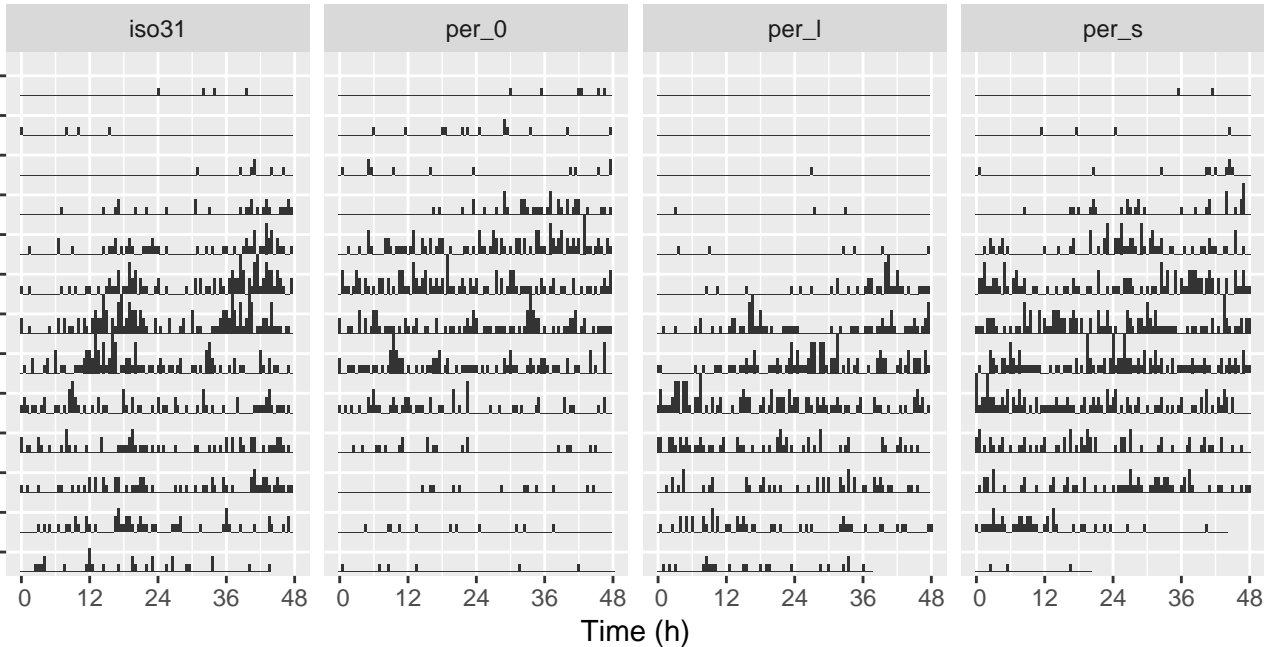

Supplement: S2 Fig — (PDF) [file pone.0331556.s002.pdf]

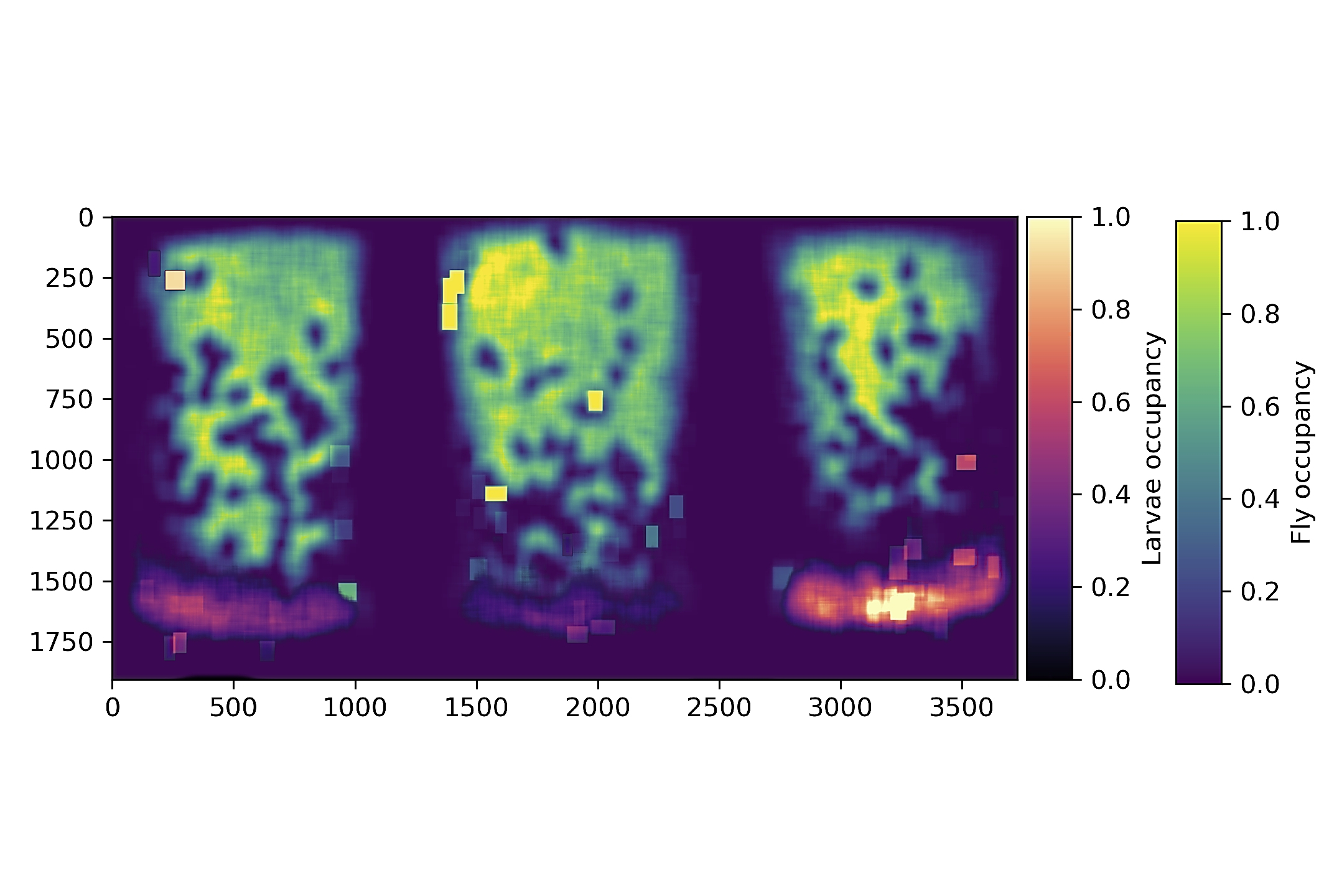

Supplement: S3 Fig — (PNG) [file pone.0331556.s003.png]

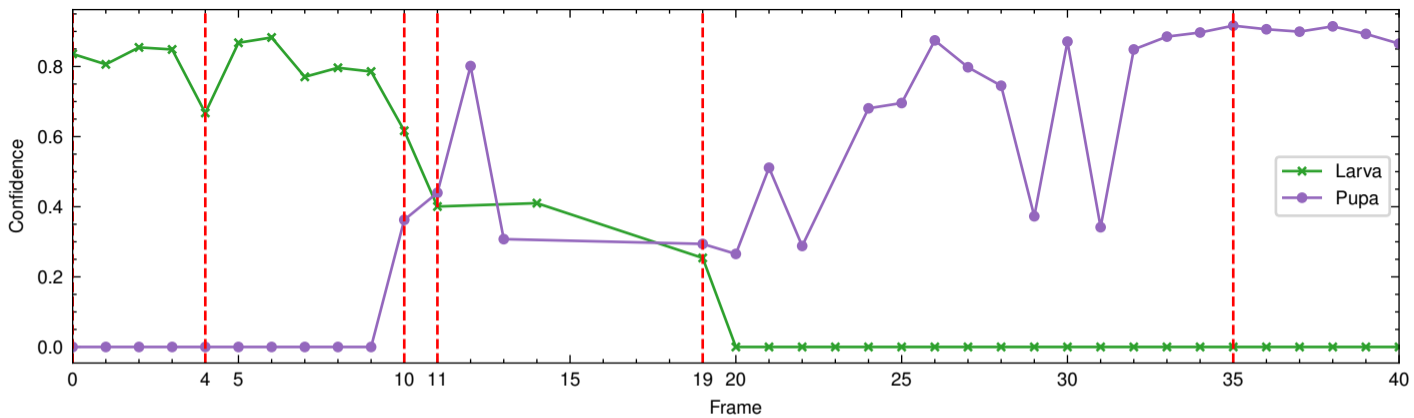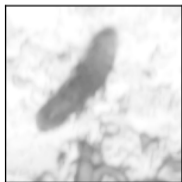

Frame 0

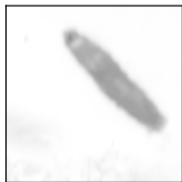

Frame 4

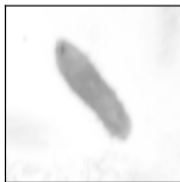

Frame 10

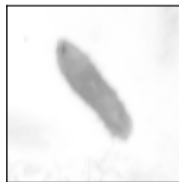

Frame 11

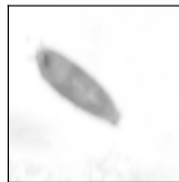

Frame 19

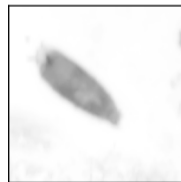

Frame 35

Supplement: S4 Fig — (PDF) [file pone.0331556.s004.pdf]

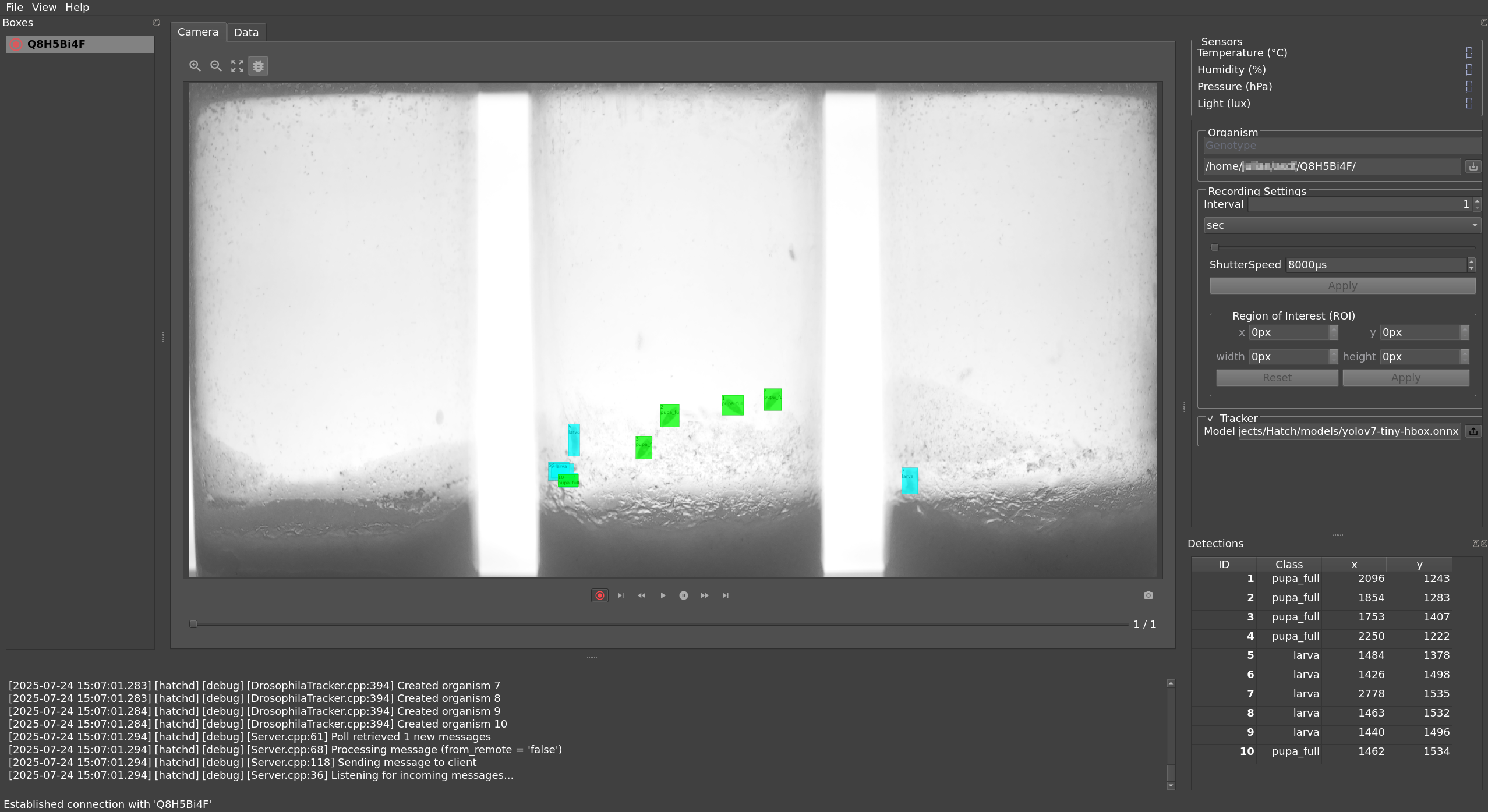

Supplement: S6 Fig — (PNG) [file pone.0331556.s006.png]
