# Supplementary material for: Hatching-Box: Automated in situ monitoring of Drosophila melanogaster development in standard rearing vials
Source: PLoS One. 2025 Sep 29;20(9):e0331556. doi: 10.1371/journal.pone.0331556 (PMC12478940; doi:10.1371/journal.pone.0331556)
Supplement: S5 Fig — (PDF) [file pone.0331556.s005.pdf]

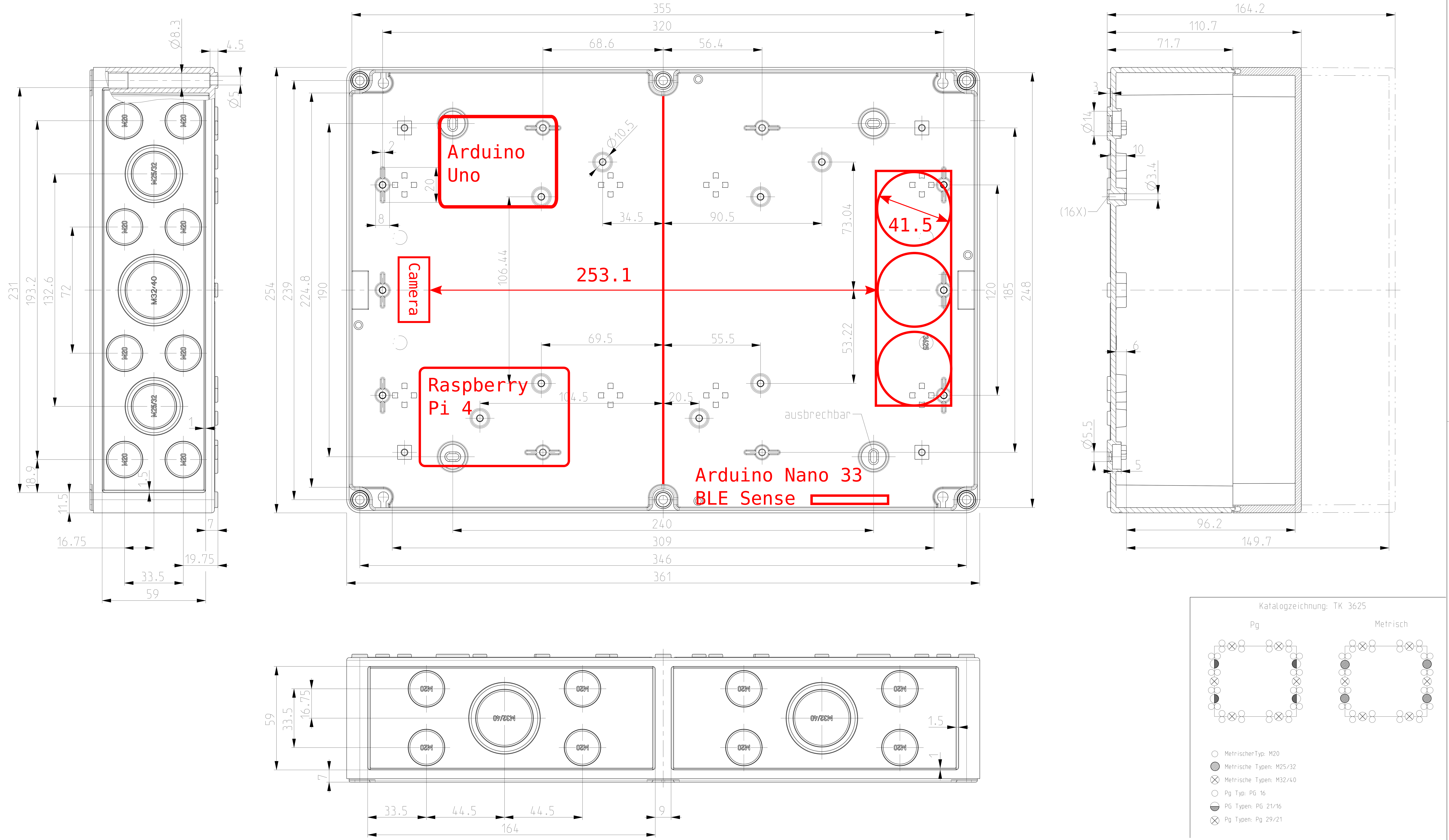

(Verwendungsbereich)		(Zul.Abw.)	(Oberflaeche)	Maßstab 1:1	(Volumen in cm³)
				(Werkstoff,Halbzeug)	
Weitergabe sowie Vervielfältigung dieses Dokuments, Verwertung und Mitteilung seines Inhalts sind verboten, soweit nicht ausdrücklich gestattet. Zuwiderhandlungen verpflichten zu Schadenersatz. Alle Rechte für den Fall der Patent-, Gebrauchsmuster- oder Geschmacksmustereintragung vorbehalten.			Datum / Name	(Benennung)	
		Bearb.	27.02.08 / WSP	Massblatt	
		Gepr.	29.05.12 / MGu	TK 3625 metrisch	
		Vers.			
		Status	Freigabe		
1	ÄM 074/12	29.05.12	WSP	(Zeichnungsnummer) M-TK-3625	Blatt 1 / AI Bl.
Zust.	Aenderung	Datum	Name	(Urspr.)	(Ersatz fuer:) Zeichnung v.11.07.2005(ME10)

(Dateiname Modell:) M-TK_3625-NEU (Dateiname Zeichnung:) M-TK_3625-NEU
