# Supplementary material for: Hatching-Box: Automated in situ monitoring of Drosophila melanogaster development in standard rearing vials
Source: PLoS One. 2025 Sep 29;20(9):e0331556. doi: 10.1371/journal.pone.0331556 (PMC12478940; doi:10.1371/journal.pone.0331556)
Supplement: S1 Table — (PDF) [file pone.0331556.s007.pdf]

Name	Model/Material	Vendor
**Main Compartement**		
Box	Spelsberg TK 3625 Grey	https://www.rs-online.com
3mm Acrylic base plate black matte	PERSPEX Frost Midnight S2 9221	https://www.expresszuschnitt.de
Infrared shield	LUXACRYL-IR 3mm	https://www.go-ttv.com
2x Infrared shield clamp	3D printed, black PLA	
Vial Holder	3D printed, white PLA	
4x Standoff	3D printed, black PLA	
3x M4 12mm	A 2	https://www.theo-schrauben.de
**Light Guide Panel**		
Light guide panel	Acrylic Transparent 10mm	
2x Light guide panel clamp	3D printed, black PLA	
Diffuser sheet	Lee Filter Roll 216 White	https://thomann.de
Infrared LEDs	SOLAROX LED IR 850NM IR1-60-850	https://www.led1.de
White LEDs	SOLAROX LED White	https://www.led1.de
2 core cable, 1m	RS PRO Control Cable, 2 Cores, $0.75mm^2$	https://www.rs-online.com
**Imaging**		
Camera Holder	Acrylic/3D printed	
Raspberry Pi camera HQ		https://www.reichelt.com
Basler Lens	Basler C125-0818-5M-P	https://www.rauscher.de
Basler Spacer Ring CS-Mount	5mm	https://www.rauscher.de
Basler filter adapter	6/8mm	https://www.rauscher.de
Basler lens filter	MIDOPT LP818-46	https://www.rauscher.de
1x Aluminium profile	Type B Slot 6mm, 20x20x110mm	https://aluprofile24.de
1x T-Slot Nut 6mm	A 2	https://aluprofile24.de
2x M3 (10mm max)	A 2	https://www.theo-schrauben.de
4x M2 (20mm)	A 2	https://www.theo-schrauben.de
4x M2 Nut	A 2	https://www.theo-schrauben.de
**Computing Hardware**		
Raspberry Pi 4	8 GB	https://www.reichelt.com
Arduino Uno		https://www.reichelt.com
Arduino Nano 33 BLE Sense		https://www.reichelt.com
Mosfet/Light Controller	Custom Shield/Arduino IRF520 MOSFET Driver Module	
External brigthness sensor	GY-302 BH1750	https://www.reichelt.com
USB A →USB B		https://www.rs-online.com
USB A →Mini-USB		https://www.rs-online.com
1x M4 Screw	A 2	https://www.theo-schrauben.de
8x M3 7mm	A 2	https://www.theo-schrauben.de
8x M3 Spacers 3mm	A 2	https://www.theo-schrauben.de
Ethernet Cable		https://www.rs-online.com
Network Switch		https://www.rs-online.com
