# Supplementary material for: Hatching-Box: Automated in situ monitoring of Drosophila melanogaster development in standard rearing vials
Source: PLoS One. 2025 Sep 29;20(9):e0331556. doi: 10.1371/journal.pone.0331556 (PMC12478940; doi:10.1371/journal.pone.0331556)
Supplement: S1 Appendix — (PDF) [file pone.0331556.s009.pdf]

S1 Appendix

Light guide panel design. For the light guide panel of the Hatching-Box we use 10mm thick acrylic glass with dimensions 93mm ×220mm which was engraved with a pattern of circular carvings to distribute the light homogeneously. We provide a script for automatically generating a carving pattern based on minimal diameter of the carvings (d_{min}) and their minimal distance between each other (dst_{min}) in millimeter.
