# Supplementary material for: Hatching-Box: Automated in situ monitoring of Drosophila melanogaster development in standard rearing vials
Source: PLoS One. 2025 Sep 29;20(9):e0331556. doi: 10.1371/journal.pone.0331556 (PMC12478940; doi:10.1371/journal.pone.0331556)
Supplement: S2 Appendix — (PDF) [file pone.0331556.s010.pdf]

S2 Appendix

Automatic detection of vials. Based on a captured sample image we first transform the pixelwise grayscale intensity in the image of dimensions $W \times H$ to a linear signal $I(x)$ $(0 < x < W)$ by applying a columnwise mean. We then calculate the derivative of $I(x)$ convolving the signal with the Sobel filter $S = \begin{bmatrix} -2 & 0 & -2 \end{bmatrix}$ to identify the most prominent changes in greyscale. Of the resulting values $D_I(x)$ we only keep those values for which applies

$$\hat{D}_I = \{D_I(x) | D_I(x) > \lambda \max_{0<x<W} D_I(x)\} \tag{1}$$

with $0 < x < W$ and configurable λ. We achieved best results with $\lambda = 0.1$. Values in $\hat{D}_I$ are sorted in descending order and further filtered by our prior knowledge of the object's dimensions in the image, e.g. rearing vials in the images are at least 1000 pixels wide, the gaps in between at least 200 pixels.
