# Supplementary material for: Hatching-Box: Automated in situ monitoring of Drosophila melanogaster development in standard rearing vials
Source: PLoS One. 2025 Sep 29;20(9):e0331556. doi: 10.1371/journal.pone.0331556 (PMC12478940; doi:10.1371/journal.pone.0331556)
Supplement: Attachment — Submitted filename: response-letter.pdf [file pone.0331556.s011.pdf]

Benjamin Risse (corr. author)
Computer Vision & Machine Learning Systems

Institute for Computer Science &
Institute for Geoinformatics
University of Münster

July 25, 2025

Dear Rachid Bouharroud,

We thank the reviewers for their thoughtful and constructive feedback, which has allowed us to further refine the focus and clarity of our study. Please find our detailed responses to the points raised below.

1 Applicability of our System for other Organisms and Studies

As a proof-of-concept, we decided to chose the circadian experiment with the different *per* mutations of *D. melanogaster* as there is comprehensive literature about this type of study. Even though our system was mainly designed with fruit flies in mind, generally the system is also able to monitor other species. However, depending on the dissimilarity compared to *Drosophila* of the species to study, the YOLO object detector might have to be retrained on images of the new species to accurately detect and classify the specimens. Furthermore, while we have not yet explicitly tested the system in a toxicological context, its performance in detecting overall behavioural patterns and developmental trends suggests that it should be well-suited for such experiments. The ability to monitor and quantify changes in population-level activity and development enables sensitive detection of toxicological effects. We plan to explore and validate these applications in future work.

2 Claim Regarding Whole Lifecycle Reconstruction and Focus on Eclosion Events

We appreciate the reviewer's observation that our current system does not demonstrate full reconstruction of the entire life cycle, and instead focuses on eclosion events. Even though live-long monitoring of the population in the vial is supported and objects are continuously classified into larvae, pupae and adults, we do not track complete life cycles of individual animals. To emphasis this fact and to avoid misunderstanding we revised the manuscript and adapted all claims and terminology accordingly.

Revised Focus: Our system aims to deliver an optimal trade-off between hardware complexity, usability, and feature extraction. The main goal is to enable *convenient and robust population-level activity monitoring* rather than comprehensive life history tracking for each individual. We have emphasized population development and gross behavioral and developmental trends, rather than individual trajectories in our manuscript.

To underscore this, we have added additional data visualizations in the form of *distribution heatmaps* for both larval and adult (fly) stages using our YOLO-based detection to the Supplementary Material. These maps demonstrate the system's utility for monitoring overall population activity and spatial distributions, which aligns with our intended application.

3 Larvae Tracking: Technical and Conceptual Limitations

High Framerate & Data Management: We acknowledge that real-time high-framerate tracking of larvae is currently infeasible within our system, primarily due to data storage and computational limits. While real-time (on-the-fly) processing could be a potential workaround, development and testing of such solutions are beyond the scope of the current manuscript. We have clarified in the revised text that current results are intended as a *proof-of-concept* to illustrate the system's potential, rather than as evidence for full lifecycle tracking.

Section on Larvae Tracking: In line with the above, we have also adjusted the relevant sections concerned with the tracking of larvae to more explicitly state these limitations and the proof-of-concept nature of our demonstration.

Loss of Larvae Out of Focal Plane: We agree this is an inherent limitation of single-camera systems – larvae may move out of the focal plane and be temporarily or permanently lost. Although technical improvements such as rotating vials or multiple cameras could mitigate this, our design prioritizes *simplicity and throughput* to enable high levels of parallelization. For population-level monitoring, loss of individuals may be compensated statistically by the increased number of tracked larvae. Long-term identity-preserving tracking is outside our scope. To mitigate this challenge in our current implementation, our detection model is trained to recognize and classify out-of-focus larvae as a separate class. We therefore keep track of in and out of focus objects (helps maintain total population counts) while excluding explicit classifications into pupae or larvae, which is often impossible given the inferior contrast of non-focused objects. We added this information to the manuscript.

Larvae Crawling into Food Not Covered: Thank you for pointing out this gap. We have expanded the Methods section to acknowledge that larvae may spend significant time within food, and as such are undetectable in our setting and clarified our focus on "wandering stage" larvae, as monitoring those outside the food is both practical and consistent with certain biological interests in this developmental stage.

4 Inclusion of Pupation Events

We agree that including pupation transitions would further strengthen the population-development perspective.

We included an explanation *why detection of pupation is less robust using visual data only*, as the behavioral and morphological changes are more gradual and rely on indirect proxies (e.g., immobility) in our revised manuscript. We have also included a novel figure showing a representative pupation event of a wiltype larva as captured by our system (Suppl. Fig. S4). The transition from larvae to pupae can clearly be seen, however given the visually continuous nature of this event, the exact moment of pupation is less clear than eclosion.

5 Robustness and Variability Compared to Konopka & Benzer

The reviewer notes that our eclosion period results (Fig. 5) appear less robust and "clean" than those of Konopka & Benzer. After reviewing their methods, we note that their data was acquired at a lower temporal resolution of one hour and hand-counted, inherently leading to smoother, less variable curves. Our automated, camera-based system is capable of higher temporal granularity, which, while capturing more detail, can also surface biological or technical variability previously unreported. A similar effect can be observed

when comparing the results of the WEclMon system with the TriKinetics systems (compare to Fig. 2 to in [11]).

In accordance with the approach described by Konopka & Benzer, we have revised our periodicity visualizations by aggregating the data from the 10-minute intervals provided by our system to 1-hour intervals. This adjustment enhances the clarity and statistical power of the observed periodic patterns, and results in visualizations that are more closely aligned with the originally published data, while still preserving the period lengths reported by our system. The updated visualization is attached below and can also be integrated in the supplement of our manuscript.

Furthermore, we appreciate the reviewer's suggestion to compare our automatic tracking method with manual labeling. We would like to clarify that our automatic detection algorithm was trained and validated on manually labeled eclosion events, effectively ensuring that its performance was benchmarked against manual annotations. As a result, the accuracy of our automatic method has already been directly assessed through this comparison as part of our model development process. We have now stated this more explicitly in the Dataset section of our revised manuscript.

6 Actogram/Periodogram (Fig. 5) Support for Rhythmicity Conclusions

Thank you for suggesting further analyses and clearer annotation. We have added clear information on the dates, duration, and number of vials analyzed. To facilitate interpretation, we have explored alternative visualization methods to more clearly illustrate periodicity. In particular, we have added a line graph depicting the number of eclosions across consecutive days of our experiments. Additional text has been included in the Results and Methods sections to clarify the experimental duration and conditions (flies monitored for 2 weeks post-oviposition, with eclosion events occurring from days 2–4, and after parental removal).

7 Summary

We hope these clarifications and additional analyses address the reviewers' concerns and improve the focus and rigor of our manuscript. Above all, we have revised our claims to emphasize *population-level activity monitoring* and the balance between system complexity and usability, rather than comprehensive individual tracking. Additionally, as requested by the reviewer, we also added a screenshot of our software to the Supplements.

Thank you once more for your valuable input.

Sincerely

Benjamin Risse
